# Supplementary material for: A short ncRNA modulates gene expression and affects stress response and parasite differentiation in Leishmania braziliensis
Source: Front Cell Infect Microbiol. 2025 Feb 5;15:1513908. doi: 10.3389/fcimb.2025.1513908 (PMC11841412; doi:10.3389/fcimb.2025.1513908)
Supplement: Supplementary file 1 [file DataSheet1.docx]

Supplementary Figure 1: Selected DE ncRNAs and preliminary functional characterization. (A) list of selected ncRNAs based on their genome location, size (shorter than 200nt) and differential expression between the *Leishmania* life stages (see Supplementary Table 1 for details). (B) Cell growth curves of the promastigote form of *Leishmania braziliensis* considering the growth of control parasites at 144h as 100%. The assay was performed in triplicate, and the (C) heat map graph shows cell growth and statistical analysis by multiple comparisons using the Dunnett method, considering *p*<0.05 and the standard deviation between replicates.


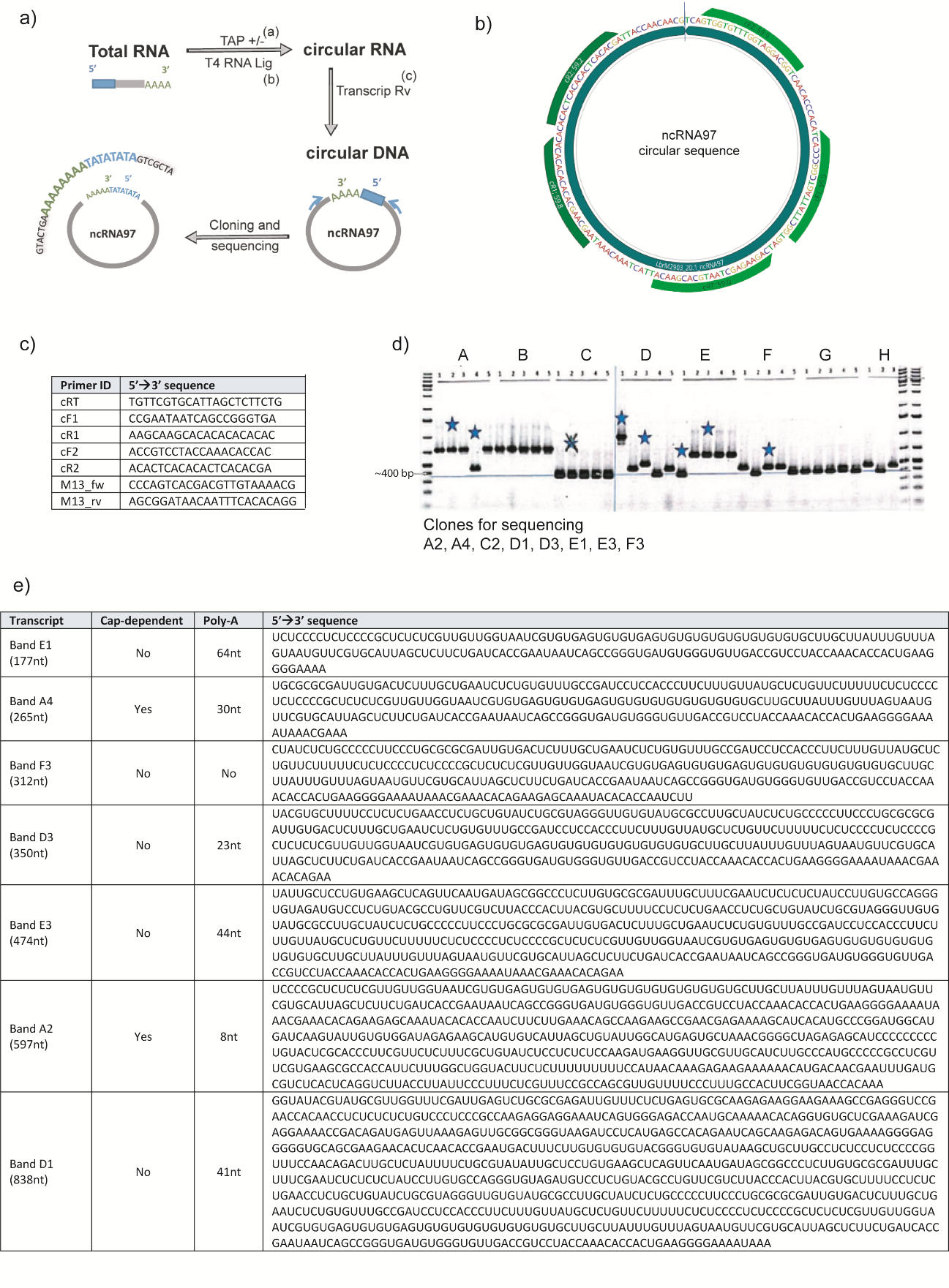


**Supplementary Figure 2.** RNA circularization assay. (a) Steps of the assay, in brief: total RNA is treated (+) or not (-) with tobacco acid pyrophosphatase (TAP) to generate monophosphate RNAs. Then, these molecules are circularized by T4 RNA ligase and reverse transcribed, releasing complementary DNA. Using specific primers, the sequence of interest is amplified by conventional PCR, cloned and inserted into a pJET plasmid and sequenced. (b) The primers cRT, cF1, cR1, cF2 and cR2 were designed based on the protocol instructions (Hang et al., 2015). (c) Sequences of primers used for the circularization assay and DNA sequencing (see Supplementary Table 2 for details). (d) Different bands were amplified after cloning, and those with divergent sizes were selected for sequencing. (e) Sequences of the different ncRNA97 transcripts found by circularization assay (see Supplementary Table 3 for details).

**
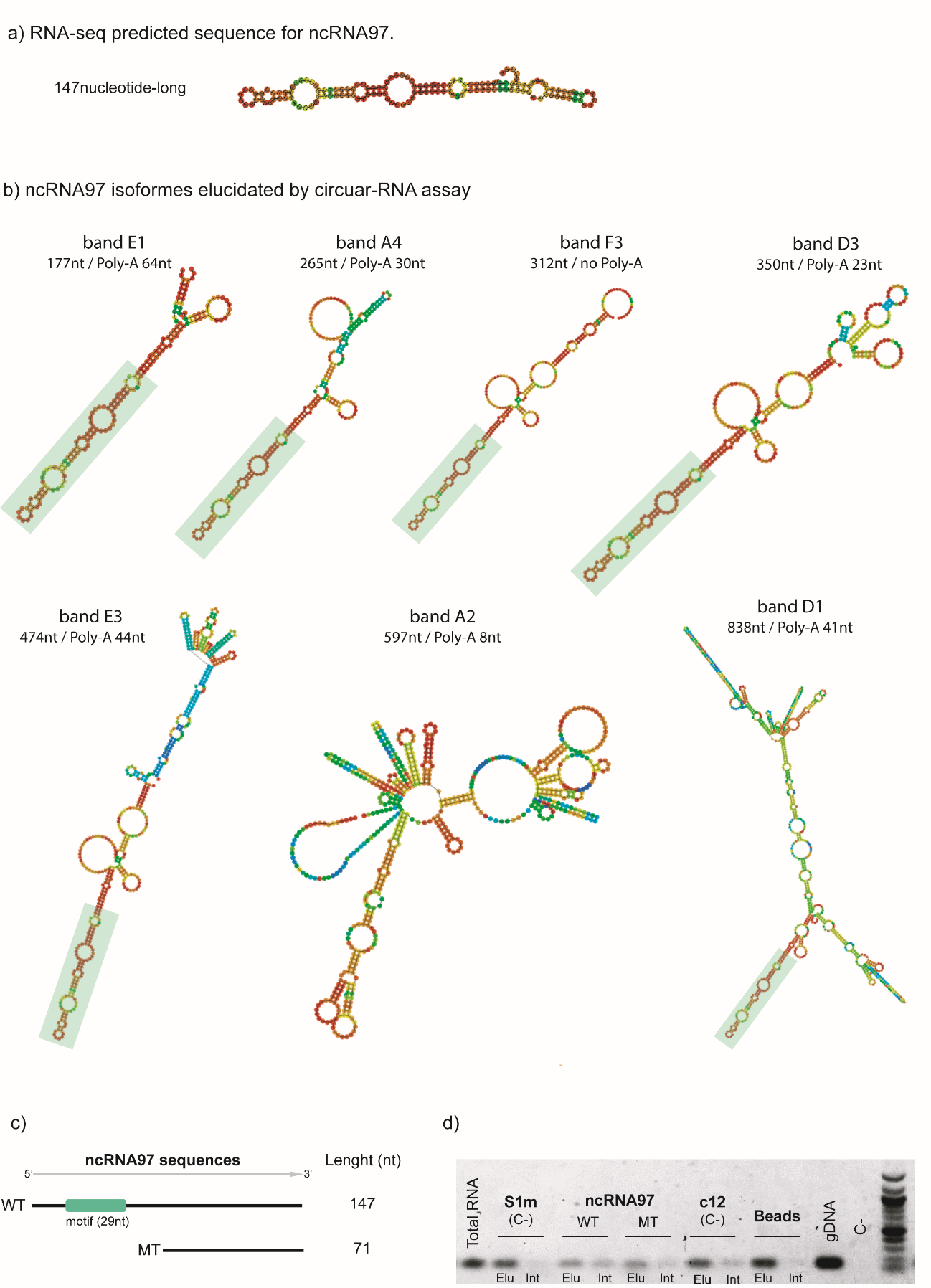
**

**Supplementary Figure 3**. Predicted sequence of ncRNA97 is conserved in six of seven *in vivo* possible isoforms. RNA from cells in the log phase was extracted and submitted to RNA circularization assay (see methods), which 7 different ncRNA97 isoforms was detected. These sequences were submitted to secondary structure prediction using RNAFold website, considering the minimal free energy (MFE) (http://rna.tbi.univie.ac.at//cgi-bin/RNAWebSuite/RNAfold.cgi).


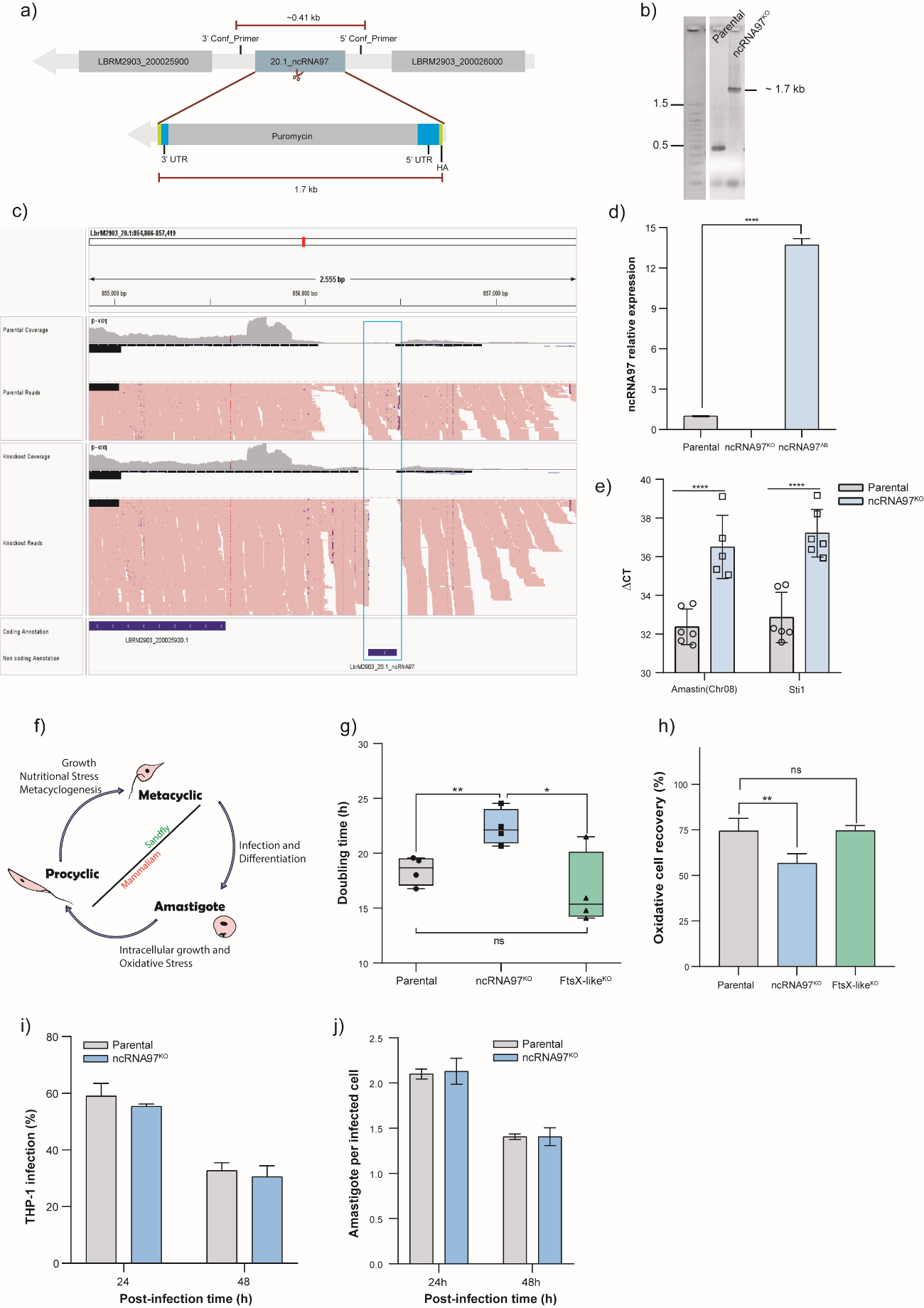


**Supplementary Figure 4**. Phenotype screening for knockout cell lines. (a) Using the CRISPR-Cas9 system, the ncRNA97 sequence was deleted and replaced with a resistance marker gene, and (b) homozygous mutants were confirmed by (b) conventional PCR and (c) transcriptomic analysis. All primers used for ncRNA97 knockout and RT‒qPCR experiments are shown in the supplementary table 2. (d) Increases in the ncRNA97 levels in the ncRNA97^AB^ parasites confirmed by RT‒qPCR. (e) ΔCT obtained by RT‒qPCR identified Amastin and stress-induced genes as downregulated in the ncRNA97^KO^ cells. (f) Based on the life cycle of *Leishmania* parasites, different assays were performed to determine the potential role of the ncRNA97 in parasites of different morphologies. (g) Differences in promastigote doubling time between the parental, ncRNA97^KO^ and Fts-X-like^KO^ cell lines. (h) ncRNA97^KO^ parasites presented lower oxidative stress tolerance after 24 h incubation in 300 µM H_2_O_2_, as demonstrated by MTT assay for cell viability. There was no difference in (i) *in vitro* TPH-1 infection or (e) intracellular amastigote growth between parental and ncRNA97^KO^ parasites.


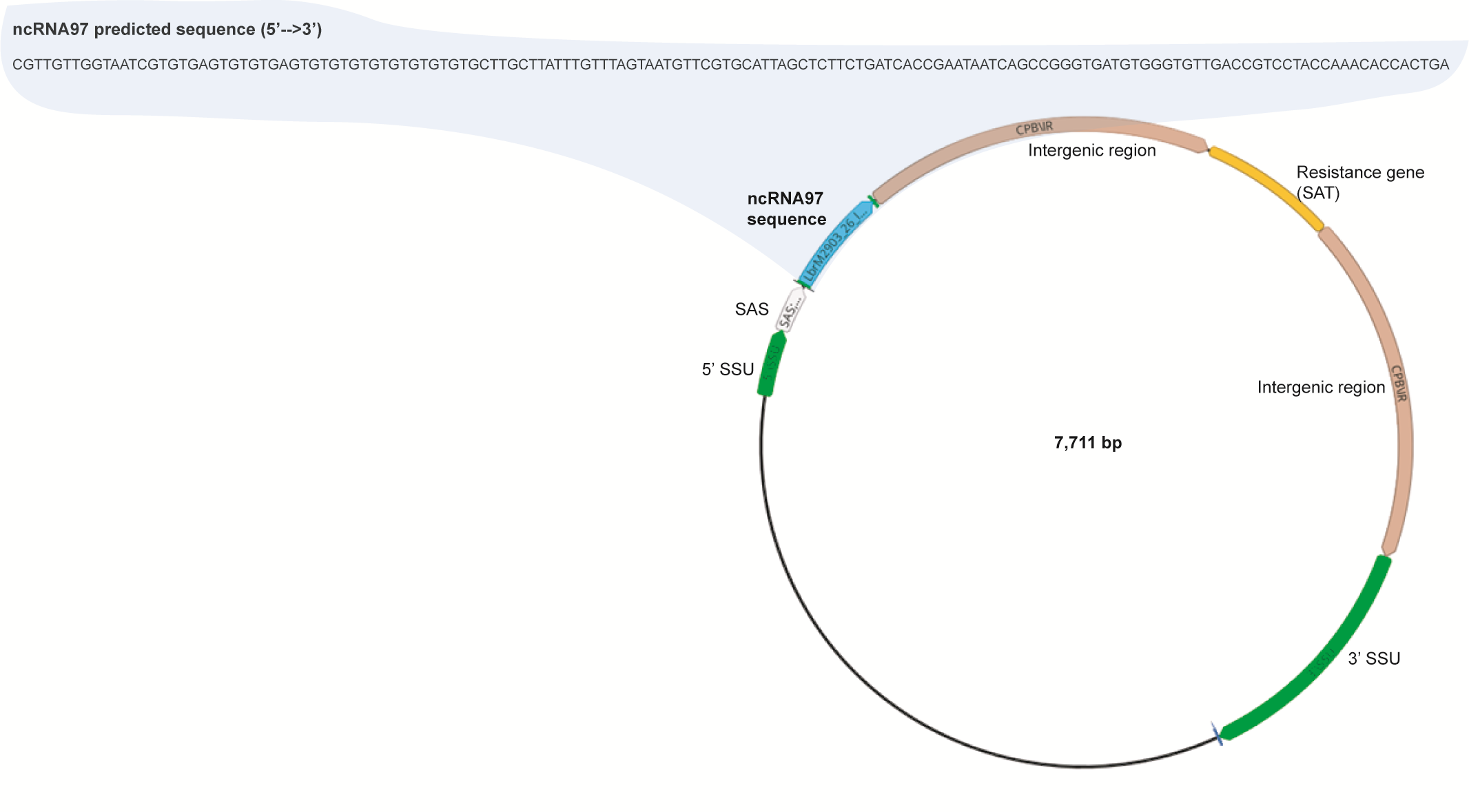


**Supplementary Figure 5**. The pSSU-SAT plasmid was used for ncRNA97^AB^ generation. The ncRNA97 sequence predicted by RNA-seq was amplified from the genomic DNA (primer sequences Fw 5’-TTGTTGGTAATCGTGTGAGT-3’, Rv 5’- AGTGGTGTTTGGTAGGACG-3’) and cloned and inserted into the plasmid using the NotI and BamHI restriction sites at the 5’ and 3’ ends, respectively. Successful molecular cloning was confirmed by DNA sequencing. ncRNA97^AB^ parasites were generated by transfecting 10^7^ ncRNA97^KO^ cells with 30 µg of the circular plasmid (no genome integration). Plasmid internalization was confirmed by PCR based on the detection of the drug resistance gene. Green: 5’ and 3’ homologous regions integrated into the linear pSSU. SAS (5’UTR): region for RNA processing/stability. The inserted ncRNA sequence is shown in blue. CPB/IR: intergenic regions of the drug resistance gene, shown in yellow (streptothricin acetyltransferase – SAT).

**Supplemental Figure 6**. Altered gene expression identified by transcriptomic analysis of parental and ncRNA97^KO^ cells. Total RNA from parental and ncRNA97^KO^ cells was extracted in biological triplicates from promastigotes on the third day of growth, treated with DNase and rRNA depletion kits and subjected to RNA sequencing (see methods section for more details). In total, 45 transcripts were differentially expressed in the KO cells compared to the parental cells (see Supplementary Table 4 for details).


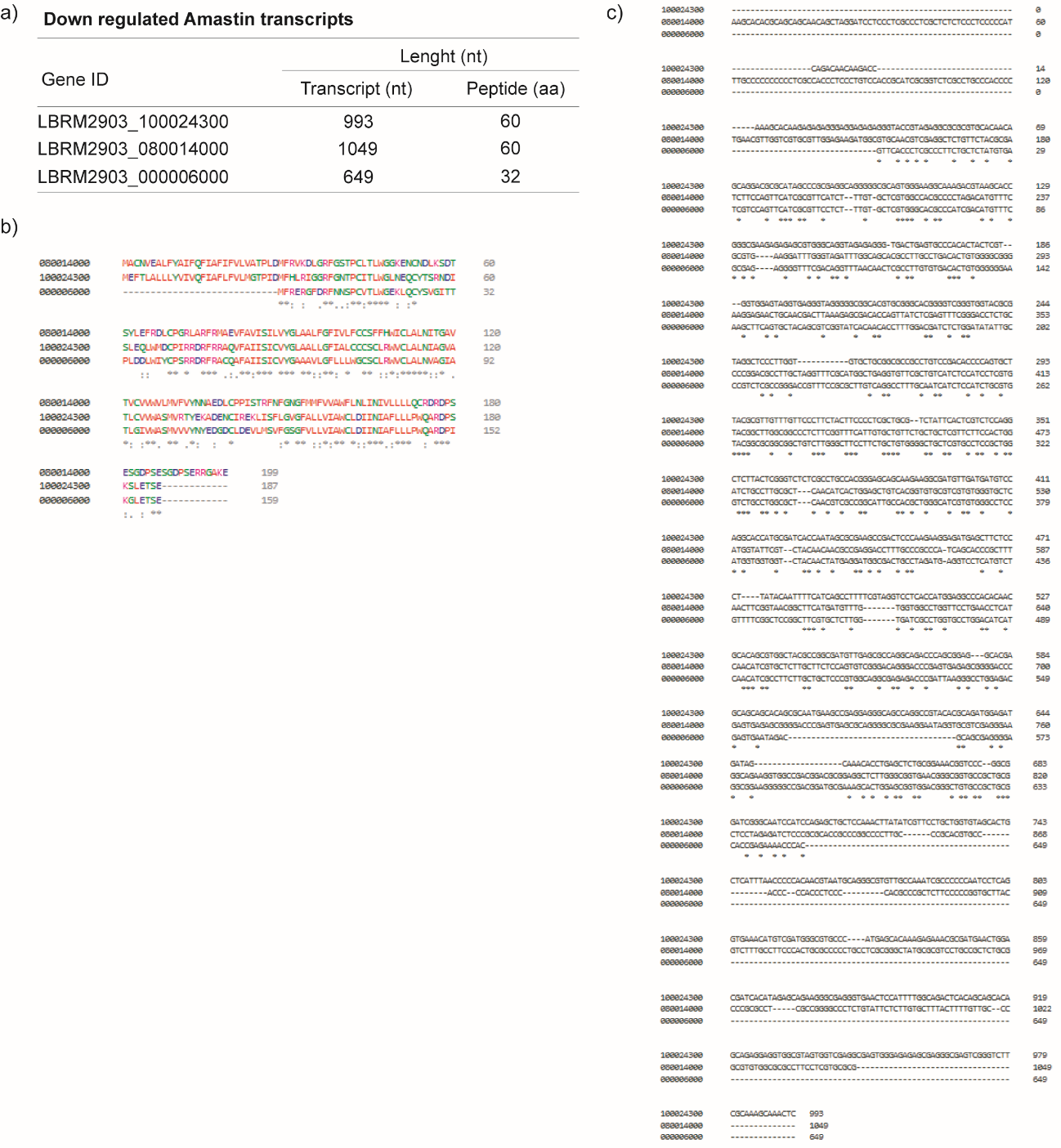


**Supplemental Figure 7**. The expression of Amastin-like protein transcripts was downregulated in ncRNA97^KO^ parasites. (A) Transcript and protein information for the downregulated genes encoding amastin-like proteins. (B) The proteins encoded by these genes are not identical, especially the LBRM2903_000006000 transcript. (C) The transcripts are also divergent in their primary sequences, in both coding and untranslated regions.


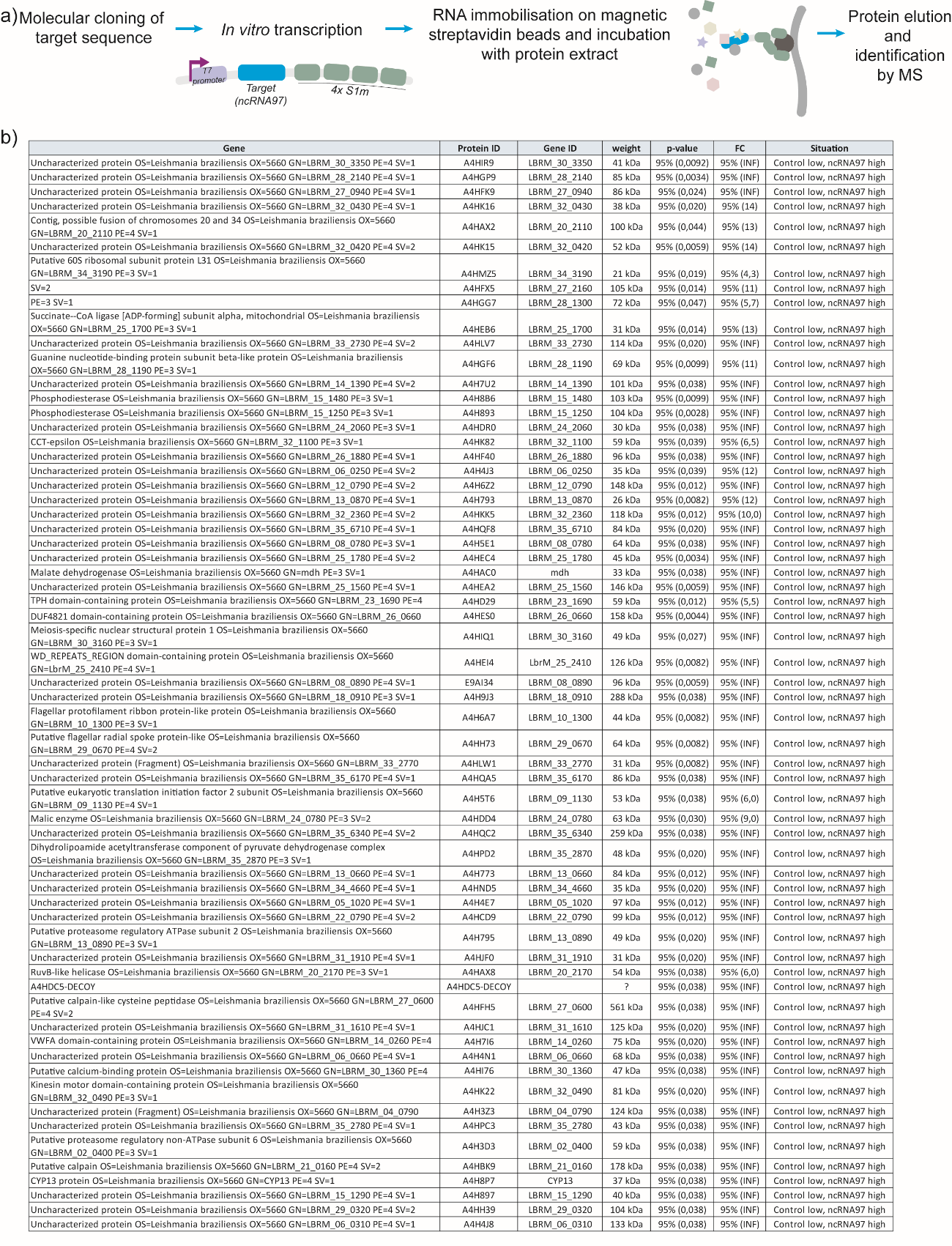


**Supplemental Figure 8**. Pulldown assay steps and protein identification. (a) An *in vitro* pulldown assay was performed as described previously (Leppek and Stoecklin, 2014) with respect to the following steps: molecular cloning, in vitro transcription, RNA immobilization on streptavidin magnetic beads, total protein extraction and incubation with immobilized RNA. RNA corresponding only to the S1m sequence was used as a negative control. Peptide identification was performed by the Proteomics Platform of the CHU de Québec Research Centre, Québec, Canada. (b) Proteins found to be enriched in the ncRNA97 samples compared to the negative control (S1m sequence only) after statistical analysis, considering *p*<0.05. The primers used for cloning the predicted ncRNA97 sequence into the plasmid used for *in vitro* transcription are described in the methods section, and PacI and NcoI were used as the restriction enzymes for the 5’ and 3’ sites, respectively (see Supplementary Table 5 for details).

**
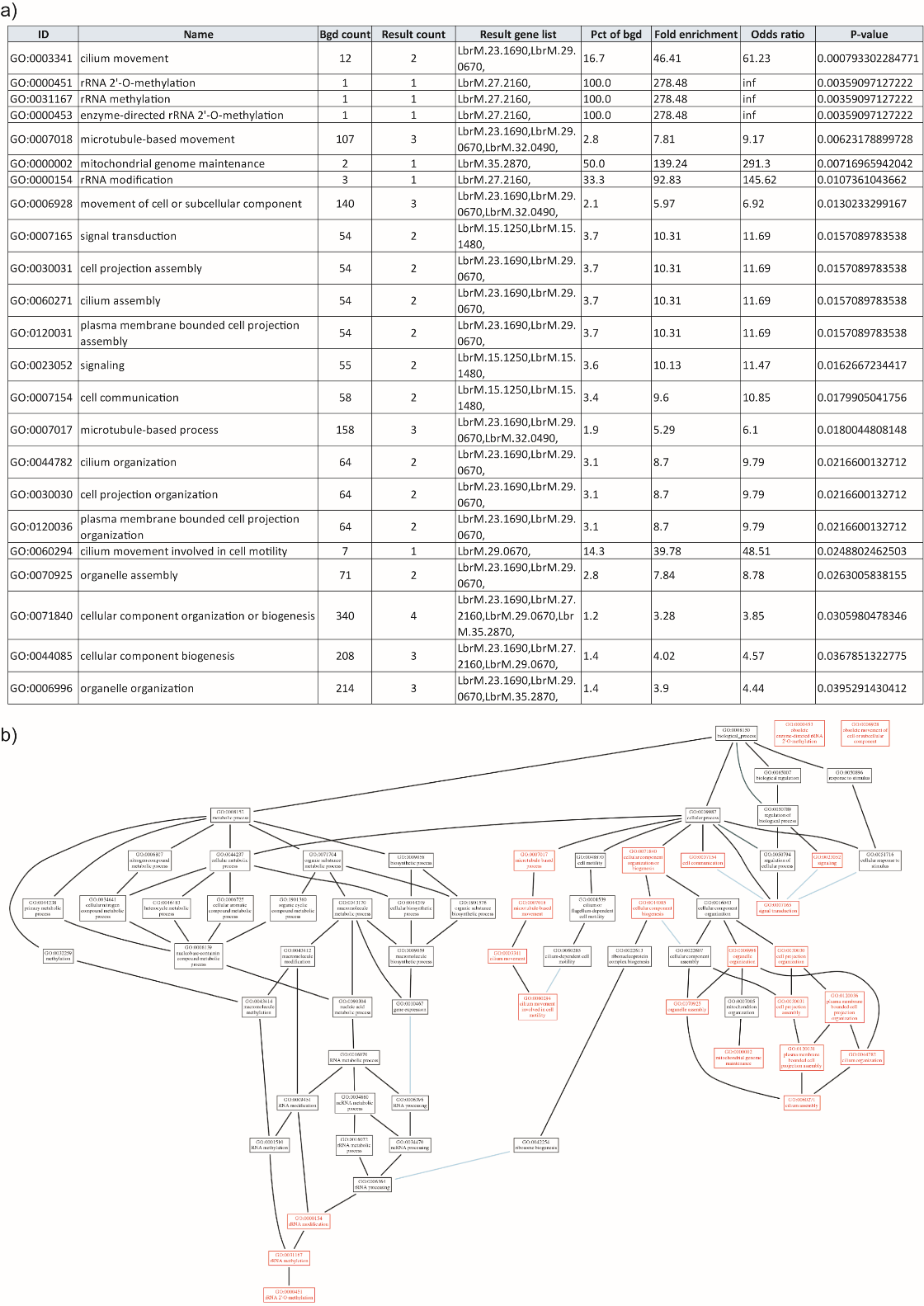
**

**Supplementary Figure 9.** Gene Ontology (GO) analysis of ncNRA97-interacting proteins. (A) GO terms found based on the proteins and their respective details. (B) GO analysis results showing the high enrichment of rRNA processing among pathways related to the ncNRA97-interacting proteins. (C) GO tree with enriched terms highlighted in red (see Supplementary Table 6 for details).
